# G6PD Enzyme Deficiency in Neonatal Pathologic Hyperbilirubinemia in Yazd 

**Published:** 2013-04-22

**Authors:** M Pahlavanzadeh, S Hekmatimoghaddam, M Teremahi Ardestani, M Ghafoorzadeh, MM Aminorraaya

**Affiliations:** 1 Doctor of Laboratory Sciences, School of Paramedicine, Shahid Sadoughi University of Medical Sciences and Health Services, Yazd, Iran.; 2 Assistant Professor of Pathology, School of Paramedicine, Shahid Sadoughi University of Medical Sciences and Health Services, Yazd, Iran.; 3 MSc in Hematology, School of Paramedicine, Bandar Abbas University of Medical Sciences, Bandar Abbas, Iran.; 4 MSc in Parasitology, School of Paramedicine, Shahid Sadoughi University of Medical Sciences and Health Services, Yazd, Iran.; 5 BSc in Laboratory Sciences, Shahid Sadoughi Hospital, Yazd, Iran.

**Keywords:** Neonatal, Jaundice, Glucosephosphate Dehydrogenase Deficiency, Bilirubin, Favism

## Abstract

**Background:**

About 7.5% of the world population carries one or two deficient copy of glucose-6-phosphate dehydrogenase (G6PD) genes. According to WHO, its prevalence in Iran is 10 to 14.9%. This study aimed on determination of frequency of G6PD deficiency in neonates with jaundice who were hospitalized during 6 months (September 2008 to February 2009) in the city of Yazd, Iran.

**Materials and Methods:**

In this study, 105 icteric neonates in the hospitals of Yazd were evaluated. Data was collected from hospital records, and the G6PD activity was measured by photometric biochemical assay. Statistical analysis of data was performed by the SPSS-16 software, using Student's t-test and Pearson's chi-squared test.

**Results:**

Between all of studied neonates, 19 (18.1%) had G6PD deficiency, and consisted of 15 boys (29.4% of boys) and 4 girls (7.4% of girls). In 100% of cases, the jaundice began in the first week after birth. The average total serum bilirubin at hospitalization was 17.22 mg/dL. In 31.5% of the G6PD-defficient neonates, exchange transfusion became necessary, which is significantly more than the rate in G6PD-sufficient (4.6%) neonates (P-value<0.05).

**Conclusion:**

In general, the frequency of G6PD deficiency in this study seems quite high. Regarding its severity and frequent need for exchange transfusion, we recommend that all of the icteric neonates should be evaluated for G6PD activity. Also, it is better to test for G6PD deficiency in all of the neonates, to detect its presence and to prevent its complications such as favism and oxidant drug-induced hemolysis, since the test has a low cost.

## Introduction

The enzyme glucose-6-phosphate dehydrogenase (G6PD) is one of the most important enzymes in the human body, present in various amounts in many cells, including red blood cells (1). Deficiency of G6PD is a frequent enzyme deficiency of the human being, with estimated 400 million affected people in the world (2). It is an inherited X-linked recessive disorder with varied clinical presentations including neonatal jaundice, hemolysis, acute icterus after exposure to chemicals and drugs, anemia, acute jaundice following consumption of fava beans (favism), and also congenital chronic non-spherocytic hemolytic anemia (3). Of these manifestations, neonatal jaundice is the earliest one, and the most critical sign for early diagnosis of this genetic disorder (4).

Around 5% of neonates with G6PD deficiency will develop jaundice after the first 24 hours of life (in contrast to fetal erythroblastosis), and their serum indirect bilirubin reaches a peak at days 3 to 5, often more than 20 mg/dL. When jaundice becomes apparent from the end of first week, its peak may be delayed up to the 2^nd^ week. Early diagnosis of deficiency of G6PD is quite important, because this disorder may cause severe hemolysis and anemia in the newborn, if undiagnosed. On the other hand, its detection is simple, rapid and cost-effective by the current laboratory methods. Although the current trend regarding G6PD deficiency in most countries is to determine the type of mutations and its relationship with other diseases or to try different therapeutic chemicals in these patients, but there is need for frequency determination in Iran in the light of genetic heterogeneity. So, we decided to find this in a 6 months period in Yazd city in central Iran.

## Materials and Methods

In this descriptive cross-sectional study, 105 icteric newborns, who were hospitalized based on pathologic hyperbilirubinemia (defined according to criteria in textbooks of pediatrics, that is, hyperbilirubinemia at the first day, direct hyperbilirubinemia, more than 5 mg/dL/d increase in serum bilirubin, hyperbilirubinemia after the day 14, or presence of any accompanying disease) and diagnosed by pediatricians in Yazd during September 2008 to February 2009 were enrolled. The questionnaire included data about demographic parameters, clinical history and lab test results. Any ambiguous or missing data was completed by calling the parents. A venous blood sample was mixed with anticoagulant EDTA, and used for G6PD enzyme activity measurement by colorimetric assay kit from Kimiapajoohan Co., Iran. In this test, 200 µL of whole blood was mixed with hemolyzing reagent, and the color development reagent was added to 200 µL of the hemolyzed blood in a vial, covered with 700 µL of liquid paraffin for inhibition of gas exchange, and incubated at 37 °C for 70 minutes. The color change in the vial was monitored continuously (at least every 15 minutes), and if this occurs before 50 minutes it was regarded as normal (adequate activity of G6PD). 

**Statistical Analysis**

In this study Student's t-test and Pearson's chi-squared test were used for comparison of means of data, using the SPSS-16 software.

## Results

The data about age of neonate at hospitalization, sex, birth weight, blood groups of neonate and mother, time of initiation of jaundice, level of total and direct serum bilirubin, and blood exchange transfusion was analyzed. The age at hospitalization was 2 to 25 days (average 14). In 100% of cases, the jaundice began in the first week after birth. Table I shows the relative frequency of G6PD deficiency in the studied neonates. 

As seen from the table, of 105 newborns, 51 (48.6%) were male and 54 (51.4%) were female. Totally, 19 neonates (18.1%) had deficiency of G6PD. The male: female ratio in icteric newborns with normal G6PD activity was about 0.7, which has a significant difference (p<0.05) with G6PD deficient newborns (in whom this ratio was 3.7). The mean serum total bilirubin at hospitalization of G6PD deficient and normal G6PD neonates was 18.43 and 11.86 mg/dL, respectively, reflecting a significant difference (p<0.05 by t-test). The average serum total bilirubin at hospitalization in all 105 neonates was 17.2 mg/dL (range 5.2-27.5). Direct bilirubin of all icteric neonates was within reference intervals (i.e., 0 to 0.2 mg/dL) at the time of hospitalization. All of the icteric neonates received phototherapy. However, 31.5% of G6PD deficient neonates and 4.6% of neonates with normal G6PD activity underwent blood exchange, again showing a significant difference (p<0.05 by chi-square test). No relationship between the relative frequency of G6PD deficiency and other assessed parameters including birth weight, gestational age, blood group of the neonate and his/her mother was found.

**Table I:** Distribution of G6PD deficiency among male and female neonates. P-value<0.05 (by chi-square test

**Sex**	**G6PD deficiency**		**Normal G6PD Activity**		**Total**
	**#**	**%**	**#**	**%**	
**Male**	36	41.9	15	78.9	51
**Female**	50	58.1	4	21.1	54
**Total**	86	100	19	100	105

## Discussion

In Asian and Mediterranean newborns, neonatal jaundice and kernicterus are more common than other regions (5). In the study by Marzban et al., the frequency of G6PD deficiency in 244 icteric neonates of Tehran, Iran, was found to be 5.7% (6). In similar studies in Hamadan, Iran (7) and in another center in Tehran, Iran, (8) the relative frequency was estimated at 4.4% and 7.6%, respectively. A study in India (9) reported that the relative frequency of G6PD deficiency in icteric neonates is 4.6%, while in Pakistan (10) it was 14.8% and in Egypt (11) it was found to be 14.4%. Deficiency of enzyme G6PD is more prevalent in Africa, Asia, Mediterranean region and Middle East, likely due to higher prevalence of infection with *Plasmodium falciparum* (12). Since the gene coding for this enzyme is located on chromosome X, it is much more common in boys, a fact which was also manifested in this study with M:F ratio being near 3.7. In the study performed in Sari, north of Iran (13), the above ratio was calculated as 3, and in another work (6) it was 3.6, both of them very close to our findings. In the current study, G6PD- deficient newborns had higher mean serum total bilirubin than other neonates (18.43 vs. 11.68 mg/dL). In a comparable study in Yasuj (14), west of Iran, the mean serum total bilirubin in G6PD-deficient newborns was 18.1 mg/dL, and in another study (15) at East Azerbaijan province, Northwest of Iran, it was 22.41 mg/dL. Since the severity of jaundice and other clinical manifestations of G6PD deficiency is directly related to the abnormal gene function and the race, it may be concluded that the prevalent mutant gene in those regions is the cause of more severe jaundice. The severe hyperbilirubinemia in neonates of our study justifies more prolonged hospitalization and more frequent use of exchange transfusion for them in comparison with neonates who had normal activity of G6PD. 

## Conclusion

In this study, G6PD enzyme deficiency in neonatal pathologic hyperbilirubinemia in Yazd was evaluated. It seems that the frequency of G6PD deficiency is quite high. Due to considerable frequency of G6PD deficiency found in this study, we suggest qualitative test of this enzyme deficiency for all icteric neonates toward early diagnosis and prevention of adverse consequences of neonatal hyperbilirubinemia, especially on brain. So, it is recommended that all of the icteric neonates should be evaluated for G6PD activity as well as to test for G6PD deficiency in all of the neonates, to detect its presence and to prevent its complications such as favism and oxidant drug-induced hemolysis, since the test has a low cost.
